# High predicted cardiac event risk in youth with obesity and type 2 diabetes: a pooled cohort analysis

**DOI:** 10.1186/s12933-025-02951-x

**Published:** 2025-10-24

**Authors:** Noemi Malandrino, Faith S. Davis, Sophia B. Glaros, Ila N. Kacker, Samson L. Cantor, Natalie A. Macheret, Geethika Thota, Lilian Mabundo, Maureen Sampson, Alan Remaley, Marissa Lightbourne, Sheela N. Magge, Jack A. Yanovski, Stephanie T. Chung

**Affiliations:** 1 Diabetes, Endocrinology, and Obesity Branch, National Institute of Diabetes and Digestive and Kidney Diseases, National Institutes of Health, 10 Center Drive, Bethesda, MD 20892 USA; 2 National Heart, Lung, and Blood Institute, 10 Center Drive, Bethesda, MD 20892 USA; 3 Division of Pediatric Endocrinology and Diabetes, Johns Hopkins University School of Medicine, 200 N. Wolfe Street, Rubenstein Bldg, Rm 3114, Baltimore, MD 21287 USA; 4 Section On Growth and Obesity, Division of Intramural Research, Eunice Kennedy Shriver National Institute of Child Health and Human Development, National Institutes of Health, 10 Center Drive, Bethesda, MD 20892 USA

**Keywords:** Youth-onset type 2 diabetes, Pediatric obesity, Cardiac event, Risk, Heart disease

## Abstract

**Background:**

Despite the growing burden of youth-onset type 2 diabetes (Y-T2D), the long-term risk for fatal/non-fatal cardiovascular disease (CVD) in Y-T2D compared to peers is unknown. The International Childhood Cardiovascular Cohort (i3C) combined-risk z-score is a novel tool for predicting 35-year risk of adult CVD events. In Y-T2D compared to peers (Lean and overweight/obesity [OW/OB]), we estimated predicted CVD events and evaluated the relationship of the i3C z-score with risk-enhancing factors.

**Methods:**

In a pooled cohort cross-sectional analysis of 1547 adolescents and young adults (AYA) aged 10–25 years [627 Lean, 803 OW/OB, 117 Y-T2D], i3C combined-risk z-scores and estimated hazard ratios (HR) were obtained from the published i3C equation using risk z-scores of systolic blood pressure, body mass index (BMI), smoking history, total cholesterol, and triglycerides. ANCOVA regression models were used: 1) to compare i3C z-scores and HR in AYA with Y-T2D, OW/OB and Lean peers, and 2) to measure associations between i3C estimated HR and risk-enhancing factors including apolipoprotein B (ApoB), total low density lipoprotein particle number (LDL-P), and high sensitivity C reactive protein (hsCRP). Models were adjusted for diagnosis group, race, study center and multiple comparisons with Bonferroni.

**Results:**

Y-T2D had the highest i3C z-score (Y-T2D: 1.23 [1.10, 1.36] vs. OW/OB: 0.84 [0.80, 0.88] vs. Lean: -0.11 [-0.15, -0.06], mean[95%CI]) and estimated HR for predicted CVD events (Y-T2D: 4.25 [3.65–4.86] vs. OW/OB: 3.04 [2.85–3.22] vs. Lean: 0.95 [0.74–1.17], HR [95% CI]). Risk-enhancing factors increased the HR for predicted CVD risk by 0.3 for each 10 mg/dL increase in ApoB, 0.1 for each 100 nmol/L increase in LDL-P, and 0.16 for each 2 mg/L increase in hsCRP, all* P* < 0.001.

**Conclusions:**

Y-T2D had an estimated 4.5- and 1.4-times higher risk for predicted CVD events compared to Lean and OW/OB peers, respectively. Lipoprotein and inflammatory risk-enhancing factors may help stratify and guide primary prevention strategies in high-risk AYA.

**Graphical Abstract:**

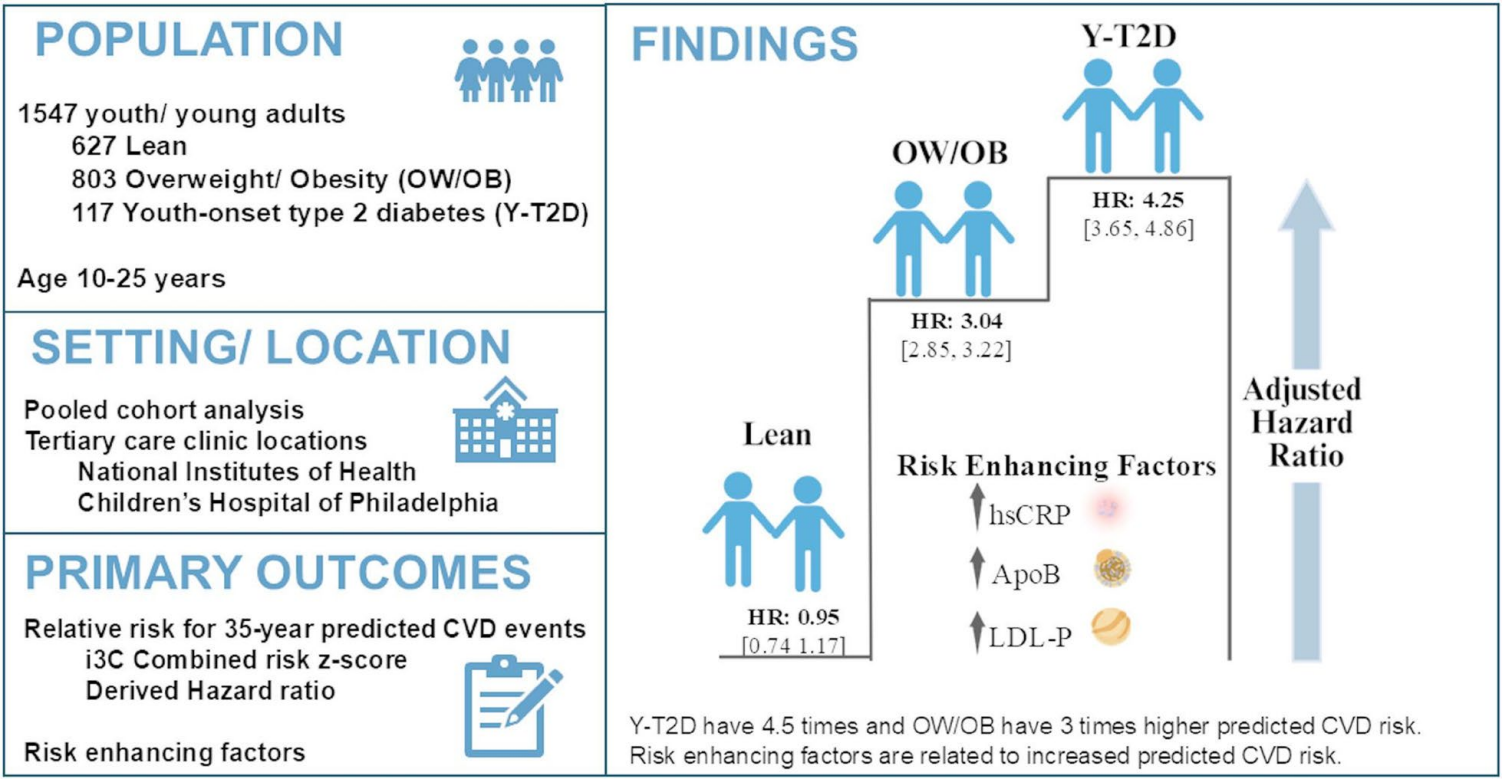

**Supplementary Information:**

The online version contains supplementary material available at 10.1186/s12933-025-02951-x.

## Research insights

**What is currently known about this topic?**

- Earlier onset of cardiovascular disease (CVD) may occur in youth-onset type 2 diabetes (Y-T2D).
- Using the i3C combined-risk model, adolescents/young adults (AYA) have 3-times higher risk for predicted CVD events per unit increase in combined-risk z-score.
- Reduction of CVD risk is critical to prevent premature CVD mortality, however data to guide primary CVD prevention in Y-T2D are limited.

**What is the key research question?**

- What is the risk for predicted CVD events per unit increase in combined-risk z-score in Y-T2D?
- Is the risk for predicted CVD events related to risk-enhancing factors in AYA?

**What is new?**

- The combination of traditional CVD risk factors within the i3C combined-risk model is associated with 4.5- and 1.4-times higher risk for predicted CVD events in Y-T2D compared to peers who are lean or with overweight/obesity respectively.
- Lipoprotein and inflammatory risk-enhancing factors LDL-P and hsCRP are strongly related to CVD event risk in AYA.

**How might this study influence clinical practice?**

- Estimating predicted CVD events via the i3C risk model and assessing CVD risk-enhancing factors may improve risk stratification to guide preventative and treatment strategies for CVD risk reduction in AYA.

## Background

Youth-onset type 2 diabetes (Y-T2D) is a rapidly progressive disease. Recent data from the SEARCH for Diabetes in Youth study have shown that, in the United States, Y-T2D may now account for up to 50% of new cases of youth-onset diabetes in adolescents and young adults (AYA) aged 15–19 years, depending on race and ethnicity, compared to youth-onset type 1 diabetes (Y-T1D) [1, 2]. Alongside the rapid rise in Y-T2D prevalence, rates of diabetes-related comorbidities in youth with Y-T2D (hypertension, hyperlipidemia, and metabolic associated fatty liver disease) have steadily increased, signifying high future cardiovascular disease (CVD) risk [3, 4]. Contemporary data on cardiac events in Y-T2D are just emerging and suggest increased mortality by the third-fourth decade of life in Y-T2D, when compared to AYA with Y-T1D with similar duration of diabetes [5]. Individuals with Y-T2D may experience earlier onset of CVD due to the aggressive natural history. Compared to youth who are lean or who have obesity, Y-T2DM may have early signs of subclinical CVD, including impaired endothelial function, increased odds of greater carotid intima-media thickness and greater arterial stiffness as measured by carotid-femoral pulse wave velocity and augmentation index, which may precede the development of hypertension in youth [6–13]. However, the timing and pace of CVD development in Y-T2D is unknown and there is limited data to support prevention paradigms [6, 14, 15].

Preventing and mitigating heart disease risk among individuals with Y-T2D is timely and critical as younger age of diabetes onset portends premature mortality, with a reduction in life expectancy by 15 years for a 30-year-old with T2D compared to a person diagnosed with diabetes at 70 years [16–19]. Recently, the International Childhood Cardiovascular Cohorts (i3C) 35-year combined-risk model was developed to guide CVD risk stratification in youth and young adults aged 3 to 19 years old [20, 21]. The i3C Consortium integrates data from seven longitudinal cohort studies designed to investigate the natural history of CVD from early childhood into adulthood. By harmonizing data across cohorts, prospective analyses demonstrated that the presence of traditional CVD risk factors during childhood, including body mass index, systolic blood pressure, total cholesterol, triglyceride levels, and smoking history, was significantly associated with fatal and non-fatal CVD events occurring before 60 years of age. Utilizing these data, the Consortium derived a composite risk z-score and corresponding hazard ratio to quantify the risk for CVD events during adulthood. Compared to youth with a z score of 0, an almost 3-times increased risk for predicted cardiac events was observed per unit increase in the combined-risk z-score [20]. However, this model has not been used in Y-T2D and the relative risk of Y-T2D compared to peers has yet to be elucidated.

Additional evidence is also needed to support CVD risk stratification in youth as traditional risk factors may be within the normative ranges during early stages of CVD. Risk-enhancing factors, such as apolipoprotein B (ApoB), low-density lipoprotein particle number (LDL-P) and high sensitivity C-reactive protein (hsCRP) are recommended for optimizing CVD risk stratification in adults, including among persons 20–39 years of age [22, 23]. Although unavailable in most clinical settings and not currently included in clinical guidelines, the use of additional biomarkers of lipoprotein insulin resistance and subclinical inflammation, such as lipoprotein insulin-resistance index (LPIR) and glycoprotein acetylation (GlycA), has also been proposed [24–26]. Yet, data are needed on whether these factors are related to predicted risk in youth.

Therefore, in a pooled cohort of AYA with Y-T2D, overweight/obesity (OW/OB), and lean (Lean) peers we compared: 1) the estimated risk for predicted fatal/non-fatal CVD events using the i3C combined-risk z-score and the i3C derived Hazard Ratio (HR); and 2) the relationship of the i3C combined-risk z-score and HR with risk-enhancing factors (ApoB, LDL-P, and hsCRP). We hypothesized that: 1) Y-T2D compared to healthy peers (both OW/OB and Lean) would have higher predicted CVD events per unit increase in combined-risk z-score and 2) incremental increases in CVD risk-enhancing factors would be related to higher predicted CVD events in all youth.

## Methods

### Overview

We conducted a pooled cohort cross-sectional secondary analysis of seventeen studies conducted at the National Institutes of Health (NIH) and the Children’s Hospital of Philadelphia (CHOP) between July 1999 and May 2024 (Supplemental Fig. 1, Supplemental Table 1). These studies were natural history protocols or studies designed to investigate behavioral or pharmacological interventions in youth and young adults with obesity and/or Y-T2D. Participants were recruited through advertisements and/or from primary care clinics and tertiary Endocrinology and Diabetes clinics. Studies were approved by their respective Institutional Review Boards, specifically at the National Institute of Diabetes and Digestive and Kidney Diseases (NIDDK), the *Eunice Kennedy Shriver* National Institute of Child Health and Human Development (NICHD), the CHOP, and the Baylor College of Medicine. Prior to study participation, written informed consent and assent were obtained.

**Table 1:** Participant Demographic and Metabolic Characteristics by Group

Variables	Total n = 1547	Lean n = 627	OW/OB n = 803	Y-T2D n = 117
Demographic Characteristics				
Age (yrs)	15.4 (15.3, 15.6)	15.4 (15.2, 15.6)	15.2 (15.1, 15.4)	16.9 (16.3, 17.5)
Biological Sex, female n(%)	1005 (65)	374 (60)	559 (70)	72 (62)
Race/Ethnicity Black White Hispanic Asian Mixed Unknown/Other	694 (45) 608 (39) 54 (3) 56 (4) 91 (6) 44 (3)	172 (27) 325 (52) 19 (3) 47 (8) 45 (7) 19 (3)	437 (55) 273 (34) 24 (3) 8 (1) 42 (5) 19 (2)	85 (73) 10 (9) 11 (9) 1 (1) 4 (3) 6 (5)
Smoking	8 (1)	0 (0)	5 (1)	3 (3)
Duration of diabetes (years)	n/a	n/a	n/a	2.3 (1.5, 3.1)
Metformin	68 (4)	0 (0)	2 (0.3)	66 (56)
Insulin	33 (2)	0 (0)	0 (0)	33 (28)
GLP-1 RA treatment	25 (2)	0 (0)	2 (0.3)	23 (20)
Anti-hypertensive treatment	15 (1)	0 (0)	0 (0)	15 (13)
Statin treatment	5 (0)	0 (0)	0 (0)	5 (4)
Metabolic Characteristics				
BMI (kg/m^2^)	30.0 (29.5, 30.5)	21.8 (21.5, 22)	35.3 (34.7, 35.9)	38.0 (36.5, 39.5)
BMI z-score	1.91 (1.80, 2.02)	0.01 (-0.04, 0.07)	3.18 (3.04, 3.32)	3.38 (3.06, 3.70)
Heart Rate (bpm)	74 (73, 75)	71 (70, 72)	76 (75, 77)	79 (76, 82)
Systolic Blood Pressure (mmHg)	118 (117, 118)	113 (112, 114)	120 (119, 121)	127 (125, 129)
Systolic Blood Pressure z-score	0.59 (0.53, 0.64)	0.13 (0.05, 0.20)	0.84 (0.77, 0.92)	1.31 (1.12, 1.51)
Diastolic Blood Pressure (mmHg)	65 (65, 66)	64 (63, 64)	66 (65, 66)	71 (69, 73)
Hemoglobin A1c (%)	5.5 (5.5, 5.6)	5.2 (5.2, 5.2)	5.4 (5.3, 5.4)	7.7 (7.3, 8.1)
Total Cholesterol (mg/dL)	156 (154, 157)	150 (148, 152)	159 (157, 161)	164 (157, 171)
Total Cholesterol z score	-0.26 (-0.31, -0.22)	-0.40 (-0.47, -0.34)	-0.17 (-0.24, -0.10)	-0.14 (-0.32, 0.05)
Triglycerides* (mg/dL)	71 (54–100)	65 (50–85)	77 (57–111)	90 (63–132)
ln(Triglycerides) z score	0.18 (0.12, 0.23)	-0.13 (-0.21, -0.06)	0.33 (0.25, 0.41)	0.77 (0.49, 1.05)
LDL Cholesterol (mg/dL)	93 (91, 94)	83 (82, 85)	99 (97, 101)	99 (93, 104)
HDL Cholesterol (mg/dL)	49 (48, 49)	54 (53, 55)	46 (45, 46)	42 (40, 44)
Non-HDL Cholesterol (mg/dL)	107 (105, 109)	97 (95, 99)	113 (111, 115)	122 (115, 129)
ApoB (mg/dL)	62 (60, 65)	50 (45, 54)	60 (57, 63)	73 (69, 78)
LPIR	35 (33, 38)	17 (13, 20)	33 (30, 36)	50 (45, 54)
LDL-P (nmol/L)	1085 (1039, 1131)	851 (776, 926)	1036 (974, 1097)	1301 (1219, 1383)
sLDL-P (nmol/L)	626 (583, 668)	467 (419, 515)	580 (528, 633)	790 (696, 884)
hsCRP* (mg/L)	1.31 (0.43–4.1)	0.17 (0.14–0.44)	1.63 (0.62–4.2)	3.3 (1.2–7.2)
GlycA (µmol/L)	390 (381, 398)	325 (311, 338)	391 (381, 402)	427 (412, 442)

Data presented as mean (95%CI), median (25th-75th percentile)*, or n(%)

Abbreviations: OW/OB: overweight/obesity; Y-T2D: youth-onset type 2 diabetes; GLP-1 RA: glucagon-like peptide 1 receptor agonist; BMI: body mass index; LDL-C: low-density lipoprotein cholesterol; Non-HDL-C: non-high-density lipoprotein cholesterol; ApoB: Apolipoprotein B; LPIR: lipoprotein insulin-resistance index; LDL-P: total low density lipoprotein particle number; sLDL-P: small low density lipoprotein particle number; hsCRP: high sensitivity C reactive protein; GlycA: glycoprotein acetylation

**Fig. 1:**
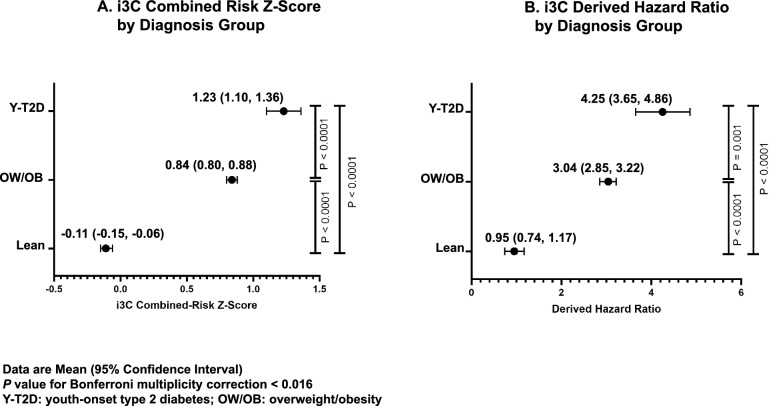
35-year i3C Combined Risk Z-Score and Derived Hazard Ratio for Predicted Cardiovascular Events Adjusted for Race and Study Center. (**A**) 35-year i3C Combined Risk Z-Score and (**B**) Derived Hazard Ratios for predicted cardiovascular events in participants with youth-onset type 2 diabetes (Y-T2D), overweight/obesity (OW/OB) and who are lean (Lean), adjusted for race and study center. i3C combined-risk z-scores were calculated as the unweighted mean of the following z-score variables: systolic blood pressure, body mass index, total cholesterol, natural logarithm of triglycerides, and smoking. Estimated hazard ratios were calculated as HR = e^Combined−Risk Z−score*1.0116^. Data are mean (95% Confidence Interval). Groups were compared with ANCOVA, adjusted for race/ ethnicity and study center, with Bonferroni correction. Y-T2D had the highest i3C combined-risk z-scores and estimated Hazard Ratios. Significant differences in the i3C combined-risk z-scores were observed in participants with Y-T2D, when compared to Lean and OW/OB. This translated to an estimated 4.5- and 1.4-times higher risk for predicted CVD events in Y-T2D per each unit increase in combined-risk z-score, compared to Lean and OW/OB. *P* value < 0.016 for Bonferroni multiplicity correction was considered statistically significant

### Study Population

Participants were 1,547 AYA aged 10–25 years old who had complete data on the five components of the i3C combined-risk z-score, and a diagnosis of Lean (n = 627), OW/OB (n = 803), or Y-T2D (n = 117) (Supplemental Fig. 1, Supplemental Table 1). Participants without diabetes were categorized by body mass index (BMI) as Lean (< 85th percentile) or overweight/obesity (OW/OB, BMI ≥ 85th percentile). Participants with Y-T2D were diagnosed by medical history, met ADA diagnostic criteria and had 2 out of 3 negative diabetes autoantibodies if GAD-65, IA-2, and insulin autoantibodies were collected, or 3 out of 4 negative antibodies if GAD-65, IA-2, insulin and ZnT-8 autoantibodies were collected (Supplemental Fig. 1) [27]. Participants taking hypoglycemic, antihypertensive and lipid-lowering medications were included in the study. Participants with other forms of diabetes or chronic systemic illnesses (e.g., type 1 diabetes or inflammatory bowel disease) were excluded. For participants who completed multiple protocols, an a priori decision was made to use the baseline visit of the first protocol. Similarly, for participants selected from intervention studies, an a priori decision was made to use data from the baseline visit. Lipoprotein and inflammatory biomarkers were available in 307 and 264 participants, respectively (Supplemental Fig. 1).

### Individual CVD risk factors and covariate assessment

Five CVD components of the i3C equation (systolic blood pressure (SBP), BMI, smoking history, total cholesterol (TC) and triglycerides (TG) concentrations) were obtained at a single visit. At each study center, one SBP measure was obtained by automated blood pressure cuff after at least 5 min of rest. Height and weight were measured three times and average values were calculated. BMI was calculated as average weight (kg) divided by average height (m) squared. Biological sex, race/ethnicity, smoking and medication history were self-reported. Smoking was categorized as “ever smoked” or “never smoked”.

Hemoglobin A1c (HbA1c) was measured by high-performance liquid chromatography (HPLC) D10 instrument (Bio-Rad, USA). Both at the NIH and CHOP, a standardized fasting lipid panel was obtained after at least an 8-h fast. In a subset of participants, lipoprotein and inflammatory biomarkers—ApoB, LPIR, total LDL-P and small LDL-P (sLDL-P), and GlycA—were measured by nuclear magnetic resonance (NMR) spectroscopy. Lipoprotein particle size and subclass concentrations were measured by the amplitudes of the lipid-methyl group NMR signals, reported in particle concentration units (nmol/L) using a 400-MHz proton NMR Profiler and Vantera Clinical Analyzer platforms [28, 29]. Partial least square regression was used to derive ApoB concentrations [30]. LPIR was calculated from a composite score of six NMR lipoprotein parameters scored 0–100, with higher score indicating higher insulin resistance [24, 31]. hsCRP was measured by immunoturbidometric method assay on Abbott Architect at the NIH.

### Recommended Thresholds

High-risk lipid panel cutoffs were defined by 2018 ACC/AHA, 2011 NHLBI/AAP and 2016 American Association of Clinical Endocrinologists and American College of Endocrinology Guidelines (Supplemental Table 2) [22, 32, 33]. High-risk hsCRP cutoffs were defined as ≥ 2 mg/L by 2019 ACC/AHA Guidelines [34].

**Table 2:** Relationship of Lipoprotein and Inflammatory Biomarkers with i3C Combined-Risk Z-Score and Derived Hazard Ratio

	i3C Combined-Risk Z-Scores				Derived Hazard Ratio			
	β	SE	Adjusted R^2^	*P-*Value	β	SE	Adjusted R^2^	*P-*Value
ApoB	0.009	0.0015	0.44	< 0.001	0.03	0.005	0.24	< 0.001
LPIR	0.01	0.0016	0.44	< 0.001	0.03	0.006	0.24	< 0.001
LDL-P	0.0004	0.00008	0.41	< 0.001	0.001	0.0003	0.20	< 0.001
sLDL-P	0.0004	0.00009	0.41	< 0.001	0.002	0.0003	0.22	< 0.001
hsCRP	0.02	0.007	0.40	< 0.001	0.08	0.02	0.20	< 0.001
GlycA	0.002	0.0005	0.39	< 0.001	0.006	0.002	0.19	< 0.001

ANCOVA regression models were used to analyze the change in i3C Combined Risk Z-Score and Derived Hazard Ratio per unit increase in Lipoprotein and Inflammatory Biomarkers, adjusted for diagnosis and race/ethnicity. A unit increase in serum levels of each of the analyzed biomarkers was associated with increased i3C combined-risk z-score and hazard ratio

β = beta-coefficient; SE: standard error. *P* value < 0.008 considered statistically significant

Abbreviations—ApoB: Apolipoprotein B; LPIR: lipoprotein insulin-resistance index; LDL-P: total low density lipoprotein particle number; sLDL-P: small low density lipoprotein particle number; hsCRP: high sensitivity C reactive protein; GlycA: glycoprotein acetylation

### Calculations

Measures of CVD risk factors were normalized to age- and sex-specific z-scores to account for age-related developmental changes. The age- and sex-specific z-scores of four CVD components of the i3C equation [SBP, BMI, TC, and natural logarithm (TG)] were generated by centering our study population measures at the i3C-Consortium’s published age- and sex-specific means, and divided by the corresponding i3C-Consortium’s standard deviations, using Eq. 1 from the original publication on the i3C prediction tool [20].

1 $$ Variable\; z\; score = ~\frac{{\left( {Observed~Participant~Value} \right) - \left( {i3C~derived~Mean} \right)}}{{\left( {i3C~derived~Standard~Deviation} \right)}} $$

Youth smoking was assigned a z-score of 2 and no smoking was assigned a z-score of 0 as previously described [20].

The combined-risk z-score was calculated as the unweighted mean of the five z-score variables, using Eq. 2, as previously prescribed [20]. A priori use of this combined-risk z-score has been proposed by the i3C Consortium to address the hypothesis that all five CVD risk factors predict future CVD events.

2 $$ i3C~Combined~Risk~z~score = ~\frac{{\begin{array}{*{20}c}  \left( {Systolic~Blood~Pressure~z~score} \right) + \left( {BMI~z~score} \right) + \left( {Smoking~z~score} \right) \\ + \left( {Total~Cholesterol~z~score} \right) + (ln\left( {Triglycerides)~z~score} \right) \\ \end{array} }}{5}$$

The estimated HR for fatal/non-fatal cardiac events was calculated using Eq. 3, as previously described [20]. This equation was derived from proportional hazard models in the i3C Consortium study, where the authors found a linear relationship between HR and z score, and an HR increase for future CVD events of 2.75 per each unit increase in combined-risk z-score during childhood. Based on these observations, Eq. (3) was developed to estimate the HR for CVD events for an individual, relative to the risk of a person with z score 0, with 1.0116 being the natural log of 2.75 [20, 21].

3 $$i3C Hazard Ratio= {e}^{Combined Risk z score *1.0116}$$

### Statistics

Demographics and participant characteristics are presented as mean (95% CI), median (IQR) or prevalence. The i3C combined-risk z scores and estimated HRs were calculated for each of the diagnosis groups, *i.e.* Lean, OW/OB and Y-T2D, as described above. Analysis of covariance was used to compare i3C combined-risk z-scores and estimated HRs among diagnosis groups, adjusting for race-ethnicity, study center (NIDDK vs. NICHD vs. CHOP), and multiple comparisons with Bonferroni. Analysis of covariance was used to study the relationship of i3C combined-risk z-scores and estimated HRs with risk-enhancing factors ApoB, LPIR, LDL-P, small LDL-P, hsCRP, and GlycA. Models were adjusted for race-ethnicity and multiple comparisons with Bonferroni. Exploratory analyses were conducted to compare the proportion of “high-risk” participants, defined as those with lipid and inflammatory markers in the recommended treatment threshold, across diagnosis groups. Chi-square or Fisher’s exact test were used, as appropriate, and multiple comparisons were adjusted using Bonferroni correction to reduce the risk of false-positive findings. Analyses were conducted with STATA (version 17.0; Stata Corp LLC). Statistical significance was inferred at a *P* value of < 0.016 for the comparison of i3C combined-risk z-scores and estimated HRs among diagnosis groups (3 diagnosis groups), and at a *P* value of < 0.008 for the relationship of i3C combined-risk z-scores and estimated HRs with risk-enhancing factors (6 risk-enhancing factors).

## Results

### Demographic and metabolic characteristics

Participant baseline characteristics are reported in Table 1. Participants were predominantly female, aged 15.4 years [95%CI: 15.3, 15.6]. Y-T2D had diabetes diagnosis for 2.3 years [95%CI: 1.5, 3.1]. Compared to OW/OB and Lean, Y-T2D were older, reported smoking, and had higher SBP, HbA1c, TG, and non-high-density lipoprotein cholesterol (non-HDL-C) concentrations. Total cholesterol and low-density lipoprotein cholesterol (LDL-C) were comparable in Y-T2D and OW/OB, and greater than in Lean. Lipoprotein biomarkers (ApoB, LPIR, LDL-P and sLDL-P) and inflammatory biomarkers (hsCRP and GlycA) were higher in Y-T2D compared to OW/OB and Lean.

Supplemental Table 2 lists the proportion of AYA who met recommended treatment thresholds. Less than 20% of Y-T2D and OW-OB had LDL-C ≥ 130 mg/dL in ‘high-risk’ category, and there was no difference between the two groups. Within the lipoprotein subgroup, a low percentage of participants had ApoB concentrations in the ‘high-risk’ range, with no difference between groups. LDL-P and hsCRP in the “risk-enhancing” range were observed in a majority of AYA with Y-T2D and OW/OB compared to Lean (*P* values for Bonferroni multiplicity correction ≤ 0.001).

### Y-T2D and OW/OB have increased predicted 35-year risk for fatal/ non-fatal cardiac events

The i3C combined-risk z-score was highest in Y-T2D in unadjusted and adjusted models (Y-T2D: 1.23 [1.10, 1.36] vs. OW/OB: 0.84 [0.80, 0.88] vs. Lean: -0.11 [-0.15, -0.06], mean[95%CI], Fig. 1A, Supplemental Fig. 2A). The HR for predicted CVD events per unit increase in combined-risk z-score was 4.5- and 1.4-times higher in Y-T2D compared to Lean and OW/OB respectively, while OW/OB had 3-times higher HR compared to Lean (Y-T2D: 4.25 [3.65–4.86] vs. OW/OB: 3.04 [2.85–3.22] vs. Lean: 0.95 [0.74–1.17], mean[95% CI], Fig. 1B, Supplemental Fig. 2B). Sensitivity analyses stratifying the population by age (greater than and less than 20 years) yielded similar results (Supplemental Table 3).

### Lipoprotein biomarkers predicted increase in cardiac events

A 10 mg/dL increase in ApoB predicted a 0.09-point increase in the i3C combined-risk z-score and 0.3-point increase in HR for 35-year predicted CVD events (*P* < 0.001; Table 2). A 10 unit increase in LPIR was associated with a 0.1-point increase in the i3C combined-risk z-score and 0.3-point increase in HR (*P* < 0.001; Table 2). Every 100 nmol/L increase in LDL-P increased the i3C combined-risk z-score by 0.04-point and 0.1-point increase in HR (*P* < 0.001; Table 2), while 100 nmol/L increase in sLDL-P predicted a 0.04-point increase in the i3C combined-risk z-score and 0.2-point increase in HR (*P* < 0.001; Table 2).

### Inflammatory biomarkers predicted increase in cardiac events

A 2 mg/L increase in hsCRP increased i3C combined-risk z-score by 0.04 points and HR by 0.16 points (*P* < 0.001; Table 2). A 100 µmol/L increase in GlycA predicted a 0.2-point increase in the i3C combined-risk z-score and a 0.6-point increase in HR (*P* < 0.001; Table 2).

## Discussion

Effective primary CVD prevention programs utilize a combination of traditional risk factors and models to predict fatal and non-fatal cardiac events, facilitating goal-directed therapy. The i3C combined risk z-score is the first comprehensive model to predict long-term CVD risk in youth and young adults. Using this model, our findings show an increased 35-year risk for predicted fatal/non-fatal CVD events in Y-T2D compared to peers with and without overweight and obesity. Predicted risk was highest in Y-T2D, despite relatively short duration of diabetes and moderately elevated HbA1c on therapy. Excess adiposity was also a major risk determinant, as youth with OW/OB also had 3-times higher risk compared to lean peers. These findings also support using a composite risk score in Y-T2D, as the combined score has been shown to be superior to individual risk factors (e.g. BMI, systolic blood pressure) for predicting future cardiac events in adulthood [20, 35]. Further, the i3C z-score strongly correlated with lipoprotein and inflammatory risk-enhancing factors (including ApoB, LDL-P and hsCRP), independent of diagnosis group and race/ethnicity. Additional studies should be directed at evaluating the predictive utility of risk-enhancing factors in young populations to refine and improve CVD risk stratification and prediction in later adulthood.

### i3C-score for CVD risk prediction in Y-T2D

To our knowledge, this is the first study to assess the i3C risk model and its relationship with risk-enhancing factors in AYA with Y-T2D. Higher CVD mortality in Y-T2D compared to peers with Y-T1D is documented in only two longitudinal registry studies. Among Australians 15–30 years, CVD-related mortality was 5-times higher in Y-T2D compared to Y-T1D of similar age, diabetes duration, and glycemic control between the two groups [5]. In the US, using the SEARCH study, adjusted standardized mortality ratio was at least 2-times higher in Y-T2D vs Y-T1D [36]. Our predicted analysis extends the literature by quantifying the risk of cardiac events using standard clinical biomarkers compared to healthy peers, emphasizing the importance of both adiposity and hyperglycemia as cardiac risk factors. Predictive equations were compared within a large, well-characterized AYA population spanning a wide range of weight and glycemic levels. The standard clinical biomarkers used in the i3C combined risk z-score are readily obtained and pragmatic for busy clinical settings, allowing for swift applicability without sacrificing time or cost. Further, using a score to predict long-term (35-year) risk would be ideal and preferable to short-term 10-year risk assessments for at-risk young adults. Short-term risk estimates are heavily dependent on age, with a greater risk of misclassification in young individuals, resulting in false reassurance in those with high lifetime risk, such as Y-T2D. Previous studies have shown a greater incidence of atherosclerotic disease and progression in at-risk young adults with low 10-year but high lifetime CVD risk, compared to those with low 10-year and low lifetime risk [37, 38]. Therefore, long‐term CVD risk scores may be better tools to guide prevention and treatment decision in young at-risk populations [39]. In our study, estimates of CVD risk in AYA were similar to those observed in the i3C cohort [20], and differences in i3C combined z-scores and hazard ratios between groups were maintained after stratification in youth compared to young adults, supporting the applicability of this predictive tool in clinical settings during emerging adulthood.

### Young age of diabetes onset and high cardiac event risk

This study adds to evidence that younger age at diabetes onset accelerates CVD progression. Our AYA cohort showed CVD risk estimates consistent with prior studies, demonstrating an inverse relationship between age and CVD risk in diabetes [16, 40, 41]. Early-onset T2D (age of diabetes onset < 40 years) more than doubles lifetime non-fatal CVD risk compared to adult-onset, partly due to longer disease duration [40, 41]. It remains uncertain whether early aggressive management of CVD risk factors during young adulthood reduces long-term adverse outcomes [42, 43]. Given the profound CVD mortality risk, universal statin therapy is recommended for primary CVD prevention in adults with T2D aged ≥ 40 years [44, 45]. In young adults 20–39 years old, guidelines recommend early statin initiation in the presence of diabetes-specific or other CVD risk-enhancers [22, 23]. No universal recommendations exist for statin initiation to prevent CVD in Y-T2D. In the presence of persistent LDL-C ≥ 130 mg/dL after 6 months of controlled glycemia, statin initiation may be considered [46]. However, achieving HbA1c < 7% is often difficult in this population. Therefore, the opportunity to benefit from statin-related CVD risk reduction in Y-T2D may be delayed several years and variable across individuals. The value of assessing risk prediction early (during adolescence and young adulthood) in this highly vulnerable group lies with the opportunity to optimize and implement early primary CVD prevention strategies. Using the i3C score risk estimates may be impactful for identifying the highest risk Y-T2D who would benefit from early statin therapy, since features of subclinical CVD have been detectable within 5 years of Y-T2D diagnosis among young adults in the absence of severe hyperglycemia and dyslipidemia [6]. Accurately assessing CVD risk and balancing with the risk for statin therapy (including teratogenic effects, myopathy) is extremely relevant in a young population of child-bearing potential. Our study provides foundational data to support clinical studies that will investigate the utility of i3C in large cohorts of Y-T2D in prevention paradigms.

### Risk-enhancing factors for improving Y-T2D CVD risk assessments

In our study, AYA with Y-T2D and OW/OB had similar increased CVD risk, despite “normal” lipid profiles. Also, established atherogenic thresholds for traditional lipid measures misclassified ~ 80% of youth with elevated i3C z-scores. The i3C Consortium reported similar findings, with future CVD risk evident at levels below current thresholds [20, 21]. Since pediatric thresholds are population-based rather than outcome-driven, additional metrics are needed to improve CVD risk stratification.

Our study provides new evidence of a strong relationship between subclinical inflammatory markers (hsCRP and GlycA) and predicted CVD event risk in young high-risk populations. Prior studies in youth have shown that both hsCRP and GlycA are associated with early subclinical signs of CVD and future risk of cardiometabolic disease, including hypertension and metabolic syndrome [24, 47]. In adults, these markers of subclinical inflammation predicted the development of T2D and CVD [26, 48, 49]. To date, the clinical value of using hsCRP as a risk-enhancing factor in youth is unproven but is supported by the fact that inflammation is a pathological feature of CVD. Targeting inflammation reduces residual CVD risk after statin treatment in adults with LDL-C levels < 70 mg/dL and elevated hsCRP [50–52]. In these individuals, using inflammatory biomarkers with predictive value for CVD risk can also aid in monitoring response to therapeutic interventions. Interestingly, several studies have suggested that hsCRP and GlycA provide information on different upstream inflammatory pathways relevant to both T2D and CVD, with GlycA possibly being a better measure of lifelong exposure to inflammatory burden and a better predictor of cardiometabolic risk in younger populations [24, 47]. In agreement with prior studies, findings from our exploratory analysis suggest that GlycA should be investigated as a tool to improve CVD risk stratification in young populations. However, GlycA is currently unavailable in most clinical settings. Whether hsCRP and GlycA may guide risk stratification of AYA with OW/OB and Y-T2D beyond traditional CVD risk factors must be further elucidated. Confirming the predictive value of these biomarkers in AYA would be particularly relevant as prior studies have shown evidence of early signs of atherosclerotic disease in young adults with Y-T2D who did not have a marked increase in traditional CVD risk factors but showed hsCRP levels in the elevated risk range [6].

LDL-C, a measure of the amount of cholesterol carried within LDL particles, is the traditional lipid marker used to estimate CVD risk and guide treatment strategies. In contrast, LDL-P is a measure of the actual number of circulating LDL particles. Although LDL-C is a strong predictor of future CVD events, elevated LDL-P has been shown to be an even stronger predictor, particularly when discordance exists between the two (i.e., low LDL-C, high LDL-P) [53, 54]. This discordance is especially prevalent among individuals with insulin resistance and associated with increased levels of atherogenic small, dense LDL particles. These particles more readily cross the arterial intima, undergo oxidation, and are taken up by macrophages to form foam cells [55]. In our study, LDL-P was elevated in the minimal to moderate-risk range in > 50% of AYA with available lipoprotein measures, despite most participants had LDL-C levels below recommended clinical thresholds. Our findings align with existing literature in youth and adult cohorts with obesity and T2D and now expand the field by demonstrating strong relationship between risk-enhancing factors and predicted cardiac event risk in AYA [28, 54]. In adults with discordantly high LDL-P, LDL-C significantly misclassifies individuals at risk for CVD, and LDL-P more accurately predicts CVD events and residual risk [54]. Our findings on the relationship between LDL-P and CVD risk in AYA are consistent with the existing literature in older individuals. Prospective studies in AYA are needed to assess whether LDL-P may aid in risk stratification and treatment decisions, resulting in prevention and/or delay of future cardiovascular disease in younger populations with cardiometabolic disease.

Apolipoprotein B is a relatively easy and accessible marker and has been shown to be a strong predictor of CVD risk, especially in adults. Thresholds are established in youth [32], but utilization is low. In this study, albeit using an ApoB derived variable in a subset of AYA, incremental increases in ApoB were associated with the highest increase in HR for predicted CVD events. However, only a small fraction of AYA who had ApoB measured would be classified as ‘high-risk’. Our findings thus are consistent with studies demonstrating an association between ApoB concentrations and predicted CVD risk but suggest that ApoB elevations may not be consistently observed in those with increased risk and more studies are needed in larger cohorts with direct ApoB measurements.

## Limitations

The i3C risk model was developed using z-scores from populations enrolled from the 1970s through the 1990s and may potentially not reflect contemporary CVD risk. The relative CVD risk may also be underestimated for Y-T2D, since youth with diabetes were not included in the development model and only a subset of individuals in the original cohorts had available measures of serum glucose. A recent study using data from a subset population of the i3C Consortium did not find a significant relationship between serum glucose z-scores in childhood and CVD risk in adulthood, but did show significantly elevated risk for CVD events at z-scores of ≥  + 0.5 for serum insulin [21]. Interestingly, back-transformation of the i3C z-scores into traditionally used measurement units showed that for all analyzed cardiometabolic risk factors, including serum insulin, the increase in relative risk for CVD events already occurred at lower levels than currently defined abnormal clinical thresholds [21]. This is particularly relevant for AYA with Y-T2D and OW/OB, who often have severe insulin resistance with compensatory hyperinsulinemia, a key pathophysiological factor in the development of atherosclerotic disease [56, 57].

Our study is a pooled cohort secondary analysis of multiple studies conducted between 1999 and 2024. Protocols performed to collect the i3C-score variables were not harmonized across studies. This may result in measurement variability due to procedural heterogeneity across study centers, potentially affecting i3C-score measures and the observed relationships with risk-enhancing factors. Nevertheless, clinical variables and laboratory tests were conducted according to standard and clinically relevant protocols. To address this bias, we included study center as a covariate in the fully adjusted model, and the findings remained robust. However, residual heterogeneity may remain.

Additional limitations include the following: 1) the recruitment of participants with Y-T2D primarily from diabetes specialty care clinics, along with a significantly larger proportion of African-American individuals compared to other racial/ethnic groups, may limit the generalizability of our findings. Although poorer glycemic control has been reported in African-American youth with T2D relative to other racial groups, prior studies have not identified significant differences in comorbidities such as dyslipidemia or hypertension, which are components of the i3C combined-risk z-score [58, 59]. Additionally, the inclusion of race/ethnicity as a covariate in our regression models did not alter the study findings. However, future studies with broader representation of youth with T2D from diverse healthcare settings and racial/ethnic backgrounds, and information on potential additional confounders, for example socio-economic status, living environment, adverse life experiences, and healthcare access, are needed for a more comprehensive assessment of the factors affecting CVD risk in AYA [60]; 2) the dual use of BMI to define participant subgroups and as a component of the i3C combined-risk z-score may introduce bias, as associations observed between BMI categories and risk for predicted CVD events may partially reflect the influence of BMI within the risk score itself. The potential value of incorporating additional measures of adiposity or metabolic health should be explored in future analyses to overcome this limitation; 3) the unequal availability of risk-enhancing measures across diagnosis groups (lowest among lean participants and highest among those with Y-T2D) may lead to an overestimation of the change in i3C z-score and derived HR per unit increase of each risk-enhancing factor; 4) due to the cross-sectional nature of our study and the limited number of participants with available risk-enhancing measures, we were unable to incorporate these variables into the i3C z-score calculation to evaluate their incremental predictive value for CVD risk. To our knowledge, risk-enhancing factors have not been consistently measured in large populations of AYA. As a result, appropriate means and standard deviations from large cohorts are not available for normalization to age- and sex-specific z-scores. Further research involving larger cohorts of AYA is warranted to evaluate the contribution of risk-enhancing factors to CVD risk prediction; 5) a relatively small sample size of Y-T2D with short diabetes duration. The narrow range of diabetes duration among our participants with YT2D limits our ability to assess the contribution of diabetes duration on predicted CVD risk. Larger cohorts with longer follow-up periods are needed to evaluate the impact of extended diabetes duration on predicted CVD risk; 6) up to 20% of participants with Y-T2D were on treatment with GLP-1 agonist. Y-T2D is still a rare condition and treatment regimens are evolving. Therefore, it is unclear how new classes of diabetes medications, such as GLP-1 agonists and sodium glucose transporter-2 inhibitors, will change CVD risk.

## Conclusions

In the presence of traditional CVD risk factors, AYA with Y-T2D or with overweight/obesity had 4.5- and 3-times higher 35-year estimated risk for predicted CVD events compared to lean peers, respectively, showing that adiposity and hyperglycemia are major determinants of projected risk in this young population. Lipoprotein and inflammatory risk-enhancing factors LDL-P and hsCRP were strongly related to CVD event risk, even when the traditional lipid profile was within normal thresholds. Utilizing the i3C risk model and risk-enhancing factors may prove useful for better identification of the highest risk Y-T2D, and to guide preventative and treatment strategies for CVD risk reduction in AYA, including early statin initiation. Future natural history studies in AYA with OW/OB and Y-T2D are needed to confirm our estimates of CVD risk, to investigate and validate the predictive potential of the i3C combined-risk z-score and risk-enhancing factors, and to evaluate whether strategies to control traditional and risk-enhancing factors at a younger age will significantly affect the progression of atherosclerotic disease and the risk for CVD events in adulthood.

## Supplementary Information

Supplementary Material 1: Supplemental Figure 1. Flowchart of participant selection - STROBE Diagram of participant selection showing inclusion and exclusion criteria and final sample size for the i3C Combined Risk z-score analysis, the lipoprotein biomarkers analysis, and the inflammatory biomarkers analysis respectively. Abbreviations: NIDDK: National Institute of Diabetes and Digestive and Kidney Diseases; NICHD: Eunice Kennedy Shriver National Institute of Child Health and Human Development; CHOP: Children’s Hospital of Philadelphia; OW/OB: overweight/obesity; Y-T2D: youth-onset type 2 diabetes; hsCRP: high-sensitivity C-reactive protein.

Supplementary Material 2: Supplemental Figure 2. Unadjusted 35-year i3C Combined Risk Z-Score and Derived Hazard Ratio for Predicted Cardiovascular Events- (A) Unadjusted 35-year i3C Combined Risk Z-Score and (B) Derived Hazard Ratios for predicted cardiovascular events in participants with youth-onset type 2 diabetes (Y-T2D), overweight/obesity (OW/OB) and who are lean (Lean). i3C combined-risk z-scores were calculated as the unweighted mean of the following z-score variables: systolic blood pressure, body mass index, total cholesterol, natural logarithm of triglycerides, and smoking. Estimated hazard ratios were calculated as HR = e^(Combined-Risk Z-score*1.0116). Data are mean (95% Confidence Interval). Groups were compared with ANCOVA with Bonferroni corrections. The i3C combined-risk z-score was highest in Y-T2D, translating to a 3.8 and 1.2-times higher risk for predicted CVD in Y-T2D compared to Lean and OW/OB respectively. The estimated risk for predicted CVD events was 3-times higher in OW/OB compared to Lean. P value < 0.016 for Bonferroni multiplicity correction was considered statistically significant.

Supplementary Material 3: Supplemental Table 1. Study Participants by Clinical Site.

Supplementary Material 4: Supplemental Table 2. Proportion of “High-Risk” Participants with Lipid Profile and Inflammatory Markers in the Recommended Treatment Threshold Per 2018 ACC/AHA; 2011 NHLBI/AAP Guidelines.

Supplementary Material 5: Supplemental Table 3. i3C Combined Risk Z-Scores and Derived Hazard Ratios Stratified by Age Group.

## Data Availability

The datasets generated and analyzed during the current study are available in the Open Science Framework repository, https://osf.io/ya3dt/.

## Abbreviations

Y-T2D: Youth-onset type 2 diabetes
AYA: Adolescents and young adults
Y-T1D: Youth-onset type 1 diabetes
CVD: Cardiovascular disease
35-year combined-risk model: International Childhood Cardiovascular Cohorts 35-year combined-risk model
ApoB: Apolipoprotein B
LDL-P: Low-density lipoprotein particle number
hsCRP: High sensitivity C-reactive protein
LPIR: Lipoprotein insulin-resistance index
GlycA: Glycoprotein acetylation
OW/OB: Overweight/obesity
Lean: Lean
HR: Hazard ratio
BMI: Body mass index
SBP: Systolic blood pressure
TC: Total cholesterol
TG: Triglycerides
HbA1c: Hemoglobin A1c
HPLC: High-performance liquid chromatography
sLDL-P: Small low-density lipoprotein particle number
NMR: Nuclear magnetic resonance
non-HDL-C: Non-high-density lipoprotein cholesterol
LDL-C: Low-density lipoprotein cholesterol
