# Supplementary material for: High predicted cardiac event risk in youth with obesity and type 2 diabetes: a pooled cohort analysis
Source: Cardiovasc Diabetol. 2025 Oct 24;24:405. doi: 10.1186/s12933-025-02951-x (PMC12551294; doi:10.1186/s12933-025-02951-x)
Supplement: Supplementary file 1 — Supplementary Material 1: Supplemental Figure 1. Flowchart of participant selection - STROBE Diagram of participant selection showing inclusion and exclusion criteria and final sample size for the i3C Combined Risk z-score analysis, the lipoprotein biomarkers analysis, and the inflammatory biomarkers analysis respectively. Abbreviations: NIDDK: National Institute of Diabetes and Digestive and Kidney Diseases; NICHD: Eunice Kennedy Shriver National Institute of Child Health and Human Development; CHOP: Children’s Hospital of Philadelphia; OW/OB: overweight/obesity; Y-T2D: youth-onset type 2 diabetes; hsCRP: high-sensitivity C-reactive protein. [file 12933_2025_2951_MOESM1_ESM.pptx]

## Slide 1
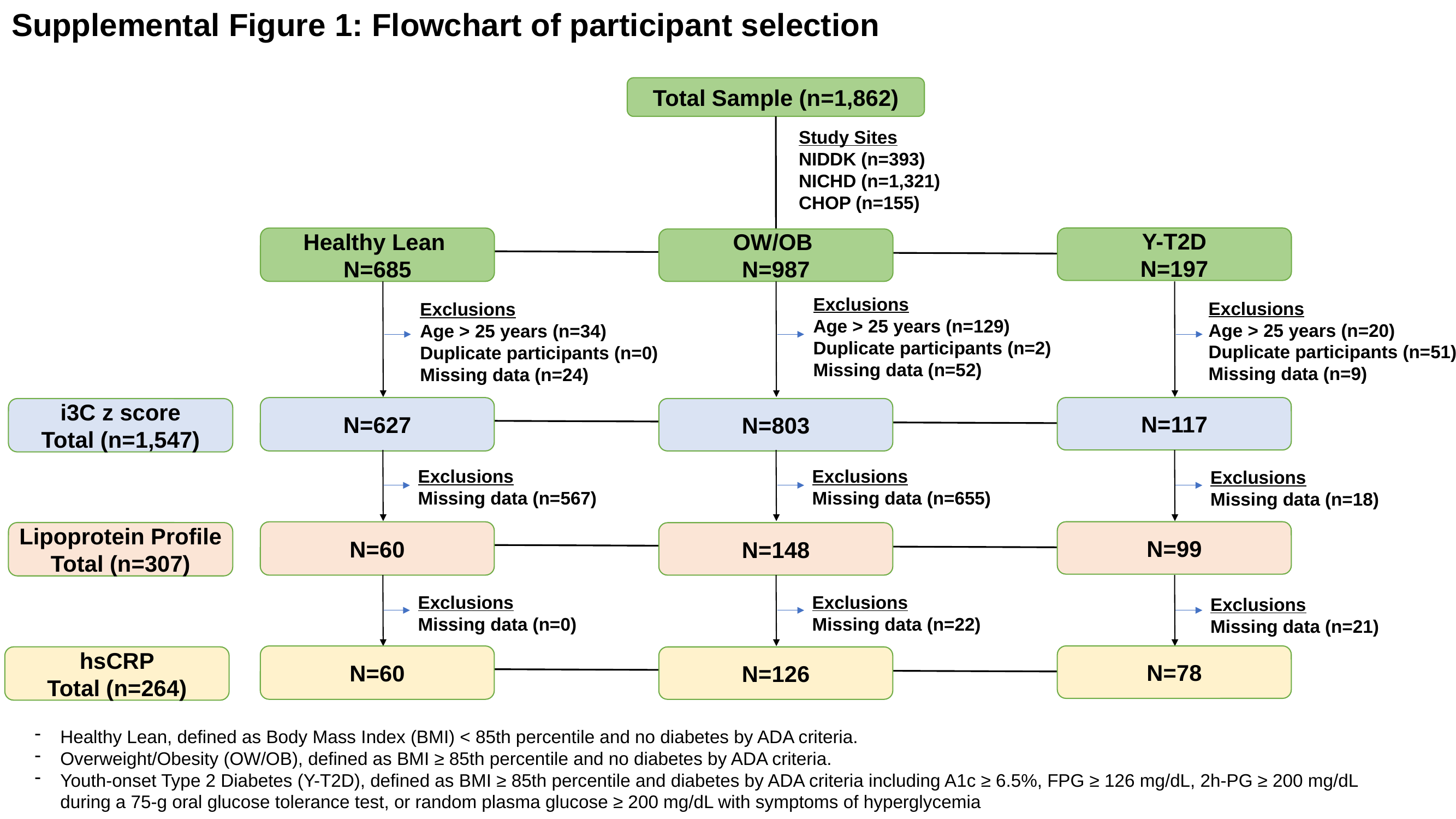

Supplemental Figure 1: Flowchart of participant selection
Total Sample (n=1,862)
Study Sites
NIDDK (n=393)
NICHD (n=1,321)
CHOP (n=155)
Y-T2D
N=197
Healthy Lean
N=685
OW/OB
N=987
Exclusions
Age > 25 years (n=129)
Duplicate participants (n=2)
Missing data (n=52)
Exclusions
Age > 25 years (n=20)
Duplicate participants (n=51)
Missing data (n=9)
Exclusions
Age > 25 years (n=34)
Duplicate participants (n=0)
Missing data (n=24)
N=117
N=627
N=803
Exclusions
Missing data (n=655)
Exclusions
Missing data (n=567)
Exclusions
Missing data (n=18)
N=99
N=60
N=148
Exclusions
Missing data (n=0)
Exclusions
Missing data (n=22)
Exclusions
Missing data (n=21)
N=78
N=60
N=126
i3C z score
Total (n=1,547)
Lipoprotein Profile
Total (n=307)
hsCRP
Total (n=264)
Healthy Lean, defined as Body Mass Index (BMI) < 85th percentile and no diabetes by ADA criteria.
Overweight/Obesity (OW/OB), defined as BMI ≥ 85th percentile and no diabetes by ADA criteria.
Youth-onset Type 2 Diabetes (Y-T2D), defined as BMI ≥ 85th percentile and diabetes by ADA criteria including A1c ≥ 6.5%, FPG ≥ 126 mg/dL, 2h-PG ≥ 200 mg/dL during a 75-g oral glucose tolerance test, or random plasma glucose ≥ 200 mg/dL with symptoms of hyperglycemia
