# Supplementary material for: High predicted cardiac event risk in youth with obesity and type 2 diabetes: a pooled cohort analysis
Source: Cardiovasc Diabetol. 2025 Oct 24;24:405. doi: 10.1186/s12933-025-02951-x (PMC12551294; doi:10.1186/s12933-025-02951-x)
Supplement: Supplementary file 2 — Supplementary Material 2: Supplemental Figure 2. Unadjusted 35-year i3C Combined Risk Z-Score and Derived Hazard Ratio for Predicted Cardiovascular Events- (A) Unadjusted 35-year i3C Combined Risk Z-Score and (B) Derived Hazard Ratios for predicted cardiovascular events in participants with youth-onset type 2 diabetes (Y-T2D), overweight/obesity (OW/OB) and who are lean (Lean). i3C combined-risk z-scores were calculated as the unweighted mean of the following z-score variables: systolic blood pressure, body mass index, total cholesterol, natural logarithm of triglycerides, and smoking. Estimated hazard ratios were calculated as HR = e^(Combined-Risk Z-score*1.0116). Data are mean (95% Confidence Interval). Groups were compared with ANCOVA with Bonferroni corrections. The i3C combined-risk z-score was highest in Y-T2D, translating to a 3.8 and 1.2-times higher risk for predicted CVD in Y-T2D compared to Lean and OW/OB respectively. The estimated risk for predicted CVD events was 3-times higher in OW/OB compared to Lean. P value < 0.016 for Bonferroni multiplicity correction was considered statistically significant. [file 12933_2025_2951_MOESM2_ESM.pptx]

## Slide 1
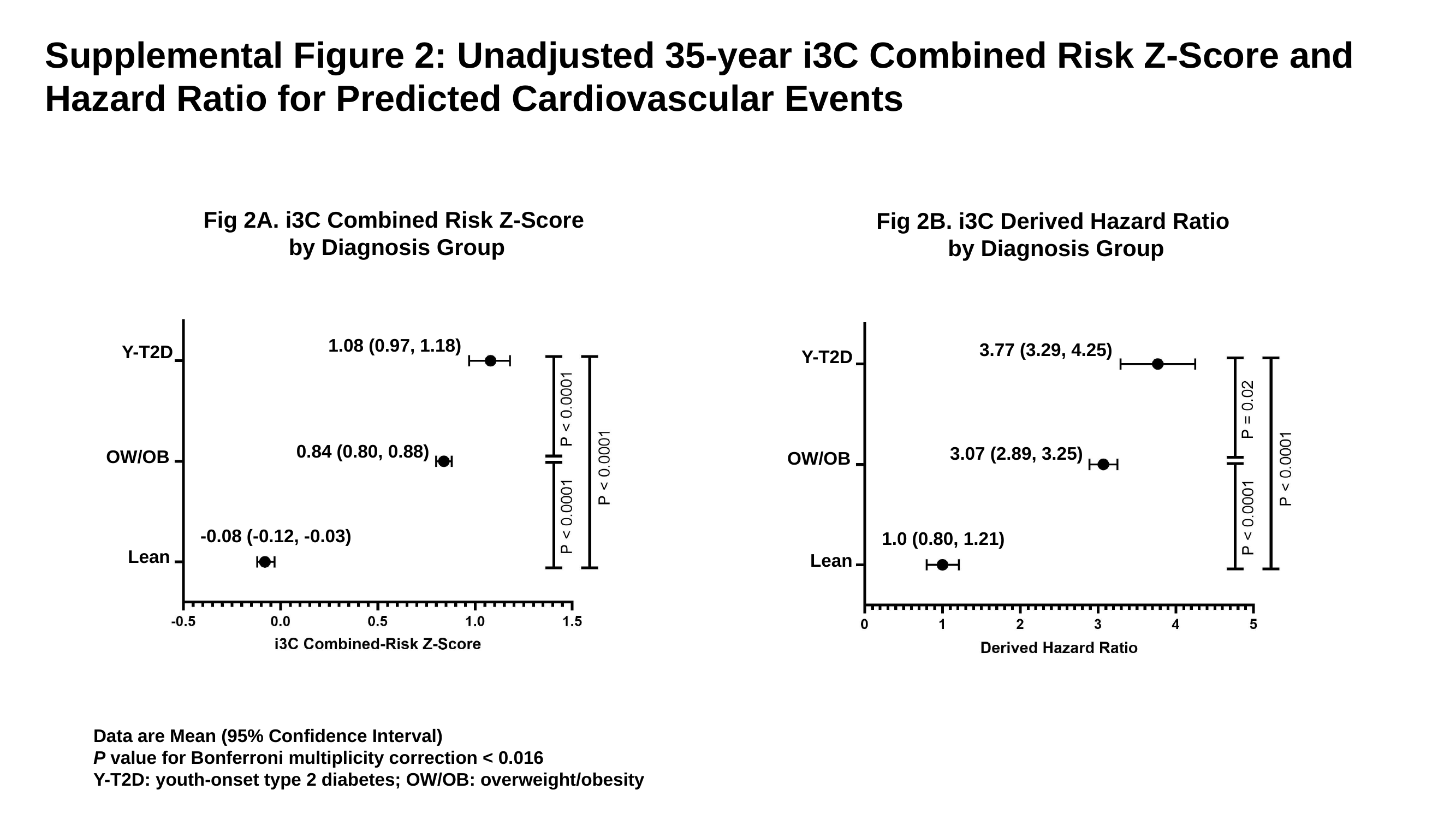

Supplemental Figure 2: Unadjusted 35-year i3C Combined Risk Z-Score and Hazard Ratio for Predicted Cardiovascular Events
Fig 2A. i3C Combined Risk Z-Score
by Diagnosis Group
Fig 2B. i3C Derived Hazard Ratio
by Diagnosis Group
1.08 (0.97, 1.18)
3.77 (3.29, 4.25)
Y-T2D
Y-T2D
0.84 (0.80, 0.88)
3.07 (2.89, 3.25)
OW/OB
OW/OB
-0.08 (-0.12, -0.03)
1.0 (0.80, 1.21)
Lean
Lean
Data are Mean (95% Confidence Interval)
P value for Bonferroni multiplicity correction < 0.016
Y-T2D: youth-onset type 2 diabetes; OW/OB: overweight/obesity
