# Supplementary material for: High predicted cardiac event risk in youth with obesity and type 2 diabetes: a pooled cohort analysis
Source: Cardiovasc Diabetol. 2025 Oct 24;24:405. doi: 10.1186/s12933-025-02951-x (PMC12551294; doi:10.1186/s12933-025-02951-x)
Supplement: Supplementary file 3 — Supplementary Material 3: Supplemental Table 1. Study Participants by Clinical Site. [file 12933_2025_2951_MOESM3_ESM.docx]

**Supplemental Table 1. Study Participants by Clinical Site**

| **Site** | **Study Clinicaltrial.gov Number** | **Number of participants** | **Diagnosis** | **% Female** | **Race** | **Age** | **BMI z score** |
| --- | --- | --- | --- | --- | --- | --- | --- |
| NIDDK  N=150 | 07-DK-0115  NCT00445627  11-DK-0168  NCT01399385  13-DK-0090  NCT01809288  16-DK-0125  NCT02830308  17-DK-0013  NCT02960659  20-DK-0018  NCT04209075  Baylor#1765 | 50  17  2  31  31  1  18 | Lean: 19 (13) OW/OB: 45 (30)  Y-T2D: 86 (57) | 94 (63) | African-American: 93 (62)  White: 16 (11)  Hispanic: 16 (11)  Asian: 7 (4)  Mixed: 9 (6)  Unknown/Other: 9 (6) | 17.5±3.4 | 2.60±2.05 |
| NICHD  n=1,244 | 96-CH-0101  98-CH-0111  04-CH-0050  08-CH-0085  NCT00631644  08-CH-0139  NCT00680979  11-CH-0239  NCT01425905  15-CH-0096  NCT02390765  16-CH-0113  NCT02769975  17-CH-0014  NCT02977403 | 187  200  111  194  107  108  279  8  50 | Lean: 567 (46)  OW/OB: 657 (53)  Y-T2D: 20 (2) | 826 (66) | African-American: 481 (39)  White: 579 (47)  Hispanic: 34 (3)  Asian: 49 (4)  Mixed: 69 (5)  Unknown/Other: 32 (2) | 15.3±2.4 | 1.78±2.26 |
| CHOP  n=153 |  | 153 | Lean: 41 (27)  OW/OB: 101 (66)  Y-T2D: 11 (7) | 85 (56) | African-American: 120 (79)  White: 13 (8)  Hispanic: 4 (3)  Asian: 0 (0)  Mixed: 13 (8)  Unknown/Other: 3 (2) | 14.6±1.4 | 2.32±1.98 |

Data are reported as n(%) or mean±SD.

Abbreviations: NIDDK - National Institute of Diabetes and Digestive and Kidney Diseases; NICHD: *Eunice Kennedy Shriver* National Institute of Child Health and Human Development; CHOP: Children’s Hospital of Philadelphia; OW/OB: overweight/obesity; Y-T2D: youth-onset type 2 diabetes
