# Supplementary material for: High predicted cardiac event risk in youth with obesity and type 2 diabetes: a pooled cohort analysis
Source: Cardiovasc Diabetol. 2025 Oct 24;24:405. doi: 10.1186/s12933-025-02951-x (PMC12551294; doi:10.1186/s12933-025-02951-x)
Supplement: Supplementary file 4 — Supplementary Material 4: Supplemental Table 2. Proportion of “High-Risk” Participants with Lipid Profile and Inflammatory Markers in the Recommended Treatment Threshold Per 2018 ACC/AHA; 2011 NHLBI/AAP Guidelines. [file 12933_2025_2951_MOESM4_ESM.docx]

**Supplemental Table 2. Proportion of “High-Risk” Participants with lipid profile and inflammatory markers in the Recommended Treatment Threshold Per 2018 ACC/AHA; 2011 NHLBI/AAP Guidelines**

| **Standard Lipid Panel** | | | | | | | | | |
| --- | --- | --- | --- | --- | --- | --- | --- | --- | --- |
|  | **Treatment Threshold** | **Total Group**  **N=1547** | **Lean**  **n=627** | **OW/OB**  **n=803** | **Y-T2D**  **n=117** | ***P*-value between groups** | ***P*-value Lean vs. OW/OB** | ***P*-value Lean vs. Y-T2D** | ***P*-value OW/OB vs. Y-T2D** |
| Total Cholesterol (mg/dL) | ≥200 | 136 (9) | 29 (5) | 87 (11) | 20 (17) | 0.0001 | 0.0001 | 0.0001 | 0.03 |
| LDL-C (mg/dL) | ≥130 | 176 (11) | 25 (4) | 133 (17) | 18 (15) | 0.0001 | 0.0001 | 0.0001 | 0.98 |
| Non-HDL-C (mg/dL) | ≥145 | 183 (12) | 28 (4) | 127 (16) | 28 (24) | 0.0001 | 0.0001 | 0.0001 | 0.01 |
| Triglycerides (mg/dL) | ≥130 | 208 (13) | 40 (6) | 140 (17) | 28 (24) | 0.0001 | 0.0001 | 0.0001 | 0.04 |
| **NMR-derived Variables** | | | | | | | | | |
|  | **Treatment Threshold** | **Total Group**  **N=307** | **Lean**  **n=60** | **OW/OB**  **n=148** | **Y-T2D**  **n=99** | ***P*-value between groups** | ***P*-value Lean vs. OW/OB** | ***P*-value Lean vs. Y-T2D** | ***P*-value OW/OB vs. Y-T2D** |
| ApoB-derived (mg/dL) | ≥110 | 12 (4) | 1 (2) | 4 (3) | 7 (7) | 0.14 |  |  |  |
| LDL-P (nmol/L) | ≥1000 | 160 (52) | 10 (17) | 74 (50) | 76 (77) | 0.0001 | 0.0001 | 0.0001 | 0.0001 |
| **Inflammatory Markers** | | | | | | | | | |
|  | **Treatment Threshold** | **Total Group**  **N=264** | **Lean**  **n=60** | **OW/OB**  **n=126** | **Y-T2D**  **n=78** | ***P*-value between groups** | ***P*-value Lean vs. OW/OB** | ***P*-value Lean vs. Y-T2D** | ***P*-value OW/OB vs. Y-T2D** |
| hsCRP (mg/L) | ≥2 | 109 (41) | 2 (3) | 55 (44) | 52 (67) | 0.0001 | 0.0001 | 0.0001 | 0.001 |

Data are reported as n (%). Between group comparisons are Chi Squared or Fisher exact test.

Abbreviations: OW/OB: overweight/obesity; Y-T2D: youth-onset type 2 diabetes; LDL-C: low-density lipoprotein cholesterol; Non-HDL-C: non-high-density lipoprotein cholesterol; ApoB: Apolipoprotein B; LDL-P: low-density lipoprotein particle number; hsCRP: high sensitivity C-reactive protein

*P value* < 0.002 for Bonferroni multiplicity correction considered statistically significant.
