# Supplementary material for: High predicted cardiac event risk in youth with obesity and type 2 diabetes: a pooled cohort analysis
Source: Cardiovasc Diabetol. 2025 Oct 24;24:405. doi: 10.1186/s12933-025-02951-x (PMC12551294; doi:10.1186/s12933-025-02951-x)
Supplement: Supplementary file 5 — Supplementary Material 5: Supplemental Table 3. i3C Combined Risk Z-Scores and Derived Hazard Ratios Stratified by Age Group. [file 12933_2025_2951_MOESM5_ESM.docx]

**Supplemental Table 3. i3C Combined Risk Z-Scores and derived Hazard Ratios Stratified by Age Group**

|  | **Age < 20y**  **n=1,441** | | **20y ≤ Age ≤ 25y**  **n=106** | |
| --- | --- | --- | --- | --- |
|  | **i3C Combined Risk z score**  **mean (95%CI)** | **i3C Hazard Ratio**  **mean (95%CI)** | **i3C Combined Risk z score**  **mean (95%CI)** | **i3C Hazard Ratio**  **mean (95%CI)** |
| Lean | -0.08 (-0.12, -0.05) | 0.99 (0.96, 1.03) | -0.002 (-0.15, 0.14) | 1.12 (0.94, 1.31) |
| OW/OB | 0.86 (0.81, 0.90) | 3.12 (2.87, 3.37) | 0.55 (0.37, 0.73) | 2.12 (1.58, 2.67) |
| Y-T2D | 1.08 (0.94, 1.21) | 3.82 (3.14, 4.49) | 1.07 (0.80, 1.35) | 3.57 (2.37, 4.76) |

Abbreviations: OW/OB: overweight/obesity; Y-T2D: youth-onset type 2 diabetes
